# Comparative Evaluation of Antimicrobial Effectiveness and Compressive Strength in Neem and Lemongrass-Modified Glass Ionomer Cement: An In Vitro Study

**DOI:** 10.7759/cureus.56234

**Published:** 2024-03-15

**Authors:** Ashwin Jaikumar Ram, Jessy Paulraj, Karthik V, Rajeshkumar Shanmugam, Subhabrata Maiti

**Affiliations:** 1 Department of Pedodontics and Preventive Dentistry, Saveetha Dental College and Hospital, Saveetha Institute of Medical and Technical Sciences, Saveetha University, Chennai, IND; 2 Nanobiomedicine Lab, Centre for Global Health Research, Saveetha Medical College and Hospital, Saveetha Institute of Medical and Technical Sciences, Saveetha University, Chennai, IND; 3 Department of Prosthodontics and Implantology, Saveetha Dental College and Hospital, Saveetha Institute of Medical and Technical Sciences, Saveetha University, Chennai, IND

**Keywords:** modified gic, compressive strength, antimicrobial, neem, lemongrass, restorative dentistry

## Abstract

Background

Glass ionomer cement (GIC) demonstrates biocompatibility and fluoride ion release, indicating their potential to inhibit a wide range of bacteria, although this remains uncertain. Lemongrass and neem are recognized for their potent antimicrobial activity against numerous pathogenic microorganisms. The objective of the study is to evaluate the antimicrobial effectiveness and compressive strength of GIC modified with neem and lemongrass.

Methodology

Lemongrass and neem were incorporated into conventional GIC at varying concentrations. Group I - neem-modified GIC (0.5%, 1%, 2%), group II - lemongrass-modified GIC (0.5%, 1%, 2%), and group III (non-modified GIC as a control group). The disk-shaped specimens were then compared to unmodified GIC (control). Antimicrobial effectiveness was assessed using the minimal inhibitory concentration (MIC) assay against *Streptococcus mutans* and *Lactobacillus*. Compressive strength was assessed using a Universal Testing Machine, with a crosshead speed set to 0.5 mm per minute. Statistical analysis was conducted with a significance level set at p < 0.05.

Results

Neem modification displayed superior antimicrobial effectiveness against both *Streptococcus mutans* and *Lactobacillus *at all concentrations when compared to the control, with 2% showing the least mean value of 0.262. In contrast, lemongrass modification exhibited a significant difference in effectiveness against *Streptococcus mutans* but no difference against *Lactobacillus*. Neem modification demonstrated superior performance compared to lemongrass (p < 0.05). Both modified groups showed no significant impact on compressive strength.

Conclusions

Neem-modified GIC demonstrated the highest antimicrobial efficacy against *Streptococcus mutans* and *Lactobacillus *without altering its compressive strength. This suggests its potential as a promising alternative material in restorative dentistry. Additional in vivo investigations are needed to assess the extended-term effectiveness of the material.

## Introduction

Early childhood caries, a prevalent chronic condition among young children, can have a detrimental impact on their health and well-being. While not life-threatening, it remains a significant concern. Efforts to identify the most effective approaches for managing and preventing dental caries are an ongoing focus of scientific investigation. Minimal intervention dentistry, a modern dental practice, is gradually gaining recognition as part of this effort [1]. The primary objective is to conserve the maximum amount of the patient’s natural tooth structure. Atraumatic restorative therapy (ART) is a notable example of how minimal intervention dentistry puts this concept into practice [2]. Using manual instruments, the process involves the removal of soft, fully demineralized carious tooth tissue as part of an alternative restorative treatment [3]. After removing the carious tissue, the cavity is restored using an adhesive dental material such as glass ionomer cement (GIC). GICs are recognized for their ability to adhere to enamel and dentin, biocompatibility, and capability to gradually release fluoride ions [4]. However, it is important to mention that in ART-treated cavities, some infected dentin may still be present as rotary burs are more effective in eliminating bacteria compared to manual instruments. Hence, secondary caries may form because cariogenic bacteria can survive beneath the GIC restoration and remain viable for up to two years [5]. Existing research suggests that the fluoride generated by GIC lacks the potency to inhibit bacterial decay effectively over an extended duration [6]. This inadequacy led to the incorporation of antimicrobial agents into GIC. Prior studies on these modifications led to changes in physical properties; hence, the effort to create an effective GIC has resulted in modifications to the current one.

The worldwide situation is evolving, with a shift toward the utilization of herbal products in the field of dentistry, often referred to as photo-dentistry [7]. Neem (scientific name *Azadirachta Indica*) is a plant belonging to the Meliaceae family, renowned for its therapeutic advantages [8]. These plant-based products are gaining increasing popularity due to their biodegradability, minimal impact on non-target organisms, cost-effectiveness, and ready availability. In Indian traditions, neem serves as a rich source of diverse medicinal compounds within traditional medicine [9]. An extract derived from *Azadirachta indica* is an herbal remedy that demonstrates antibacterial properties. Dried neem chewing sticks exhibit the highest antibacterial efficacy against *Streptococcus mutans *in comparison to other bacteria associated with dental caries [10]. Nimbidin, nimbin, nimbolide, azadirachtin, gallic acid, epicatechin, catechin, and margolone present in neem showcase robust antibacterial properties. The primary bioactive component in neem is azadirachtin, which is renowned for its antimicrobial properties [11].

Lemongrass (scientific name *Cymbopogon citratus*) is an herb that belongs to the Poaceae family. It is named for its lemon-like scent, which is attributed to the presence of the citral cyclic monoterpene. Lemongrass is rich in phytochemicals such as tannins, flavonoids, alkaloids, and a variety of essential oils. Its leaves and stems are rich in compounds such as flavonoids, tannins, geranyl acetate, phenolic compounds, steroids, terpenoids, coumarins, and saponins, which contribute to its antimicrobial, antibacterial, anti-inflammatory, and antifungal properties [12]. Furthermore, numerous research investigations have recorded the antibacterial properties of lemongrass oil against a broad range of microorganisms [13,14]. Insufficient evidence exists in the current literature regarding the use of lemongrass and neem in GIC for restorative applications. While there is established literature supporting their effectiveness in mouthwash and toothpaste, there is limited research on their use as restorative materials. Therefore, this study's objective is to examine and assess the antimicrobial efficiency and compressive strength of GIC when modified with neem and lemongrass compared to traditional GIC. The null hypothesis stated that there is no difference between traditional GIC and neem and lemongrass-modified GIC.

## Materials and methods

Ethical approval

For this in vitro study, ethical clearance was obtained from the institutional review board under the reference number SRB/SDC/UG-1876/22/PEDO/025.

Sample size

The GPower sample size calculator was used to determine the required sample size. According to the calculation, to achieve a power of 0.95 (95% confidence interval) with an effect size of 0.25 for each parameter comprising both antimicrobial activity and compressive strength, 84 samples would be required.

Materials used

The study aimed to evaluate the antimicrobial effectiveness and compressive strength of materials, including (a) bacterial strains (*Streptococcus mutans* and *Lactobacillus*), (b) dried neem leaves, (c) dried lemongrass leaves, and (d) conventional GIC sourced from GC Corporation.

Specimen preparation

**Figure 1:**
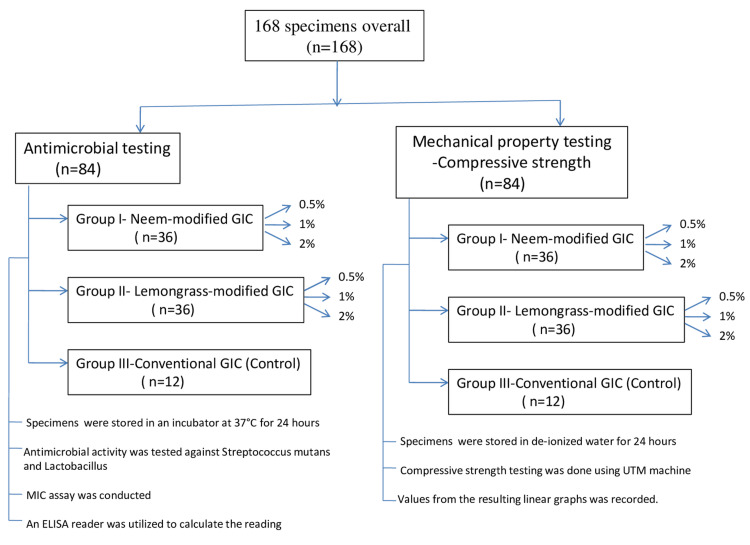
Flowchart of the study design. GIC = glass ionomer cement; MIC = minimal inhibitory concentration; ELISA = enzyme-linked immunosorbent assay; UTM = Universal Testing Machine

In this study, type IX GIC (GC Corporation) was employed. To create the experimental cement, dried neem and lemongrass leaves were powdered and added in various weight percentages (0.5%, 1.0%, and 2.0%) to the GIC powder. The experimental groups consisted of group I (neem-modified GIC with 0.5%, 1%, and 2%), group II (lemongrass-modified GIC with 0.5%, 1%, and 2%), and group III (control group with conventional GIC) (Figure 1).

**Figure 2:**
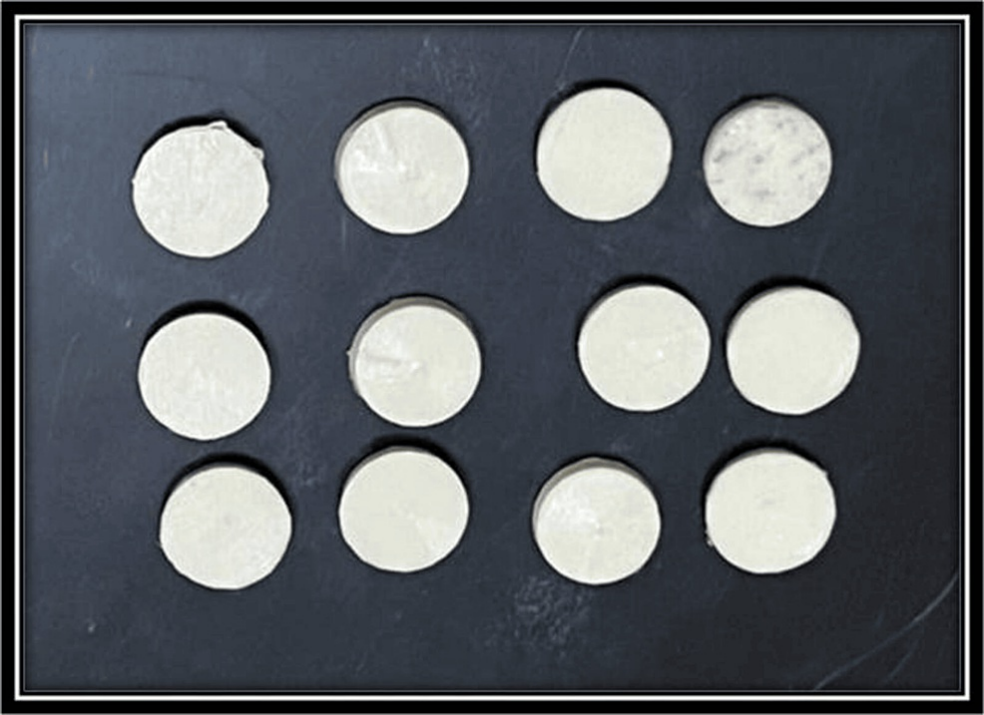
Test specimens for antimicrobial analysis.

The mixture was first manually mixed and then subjected to five minutes of vibration for thorough blending. Disk-shaped specimens with a diameter of 6 mm and a height of 2 mm were crafted for each experimental group (Figure 2).

**Figure 3:**
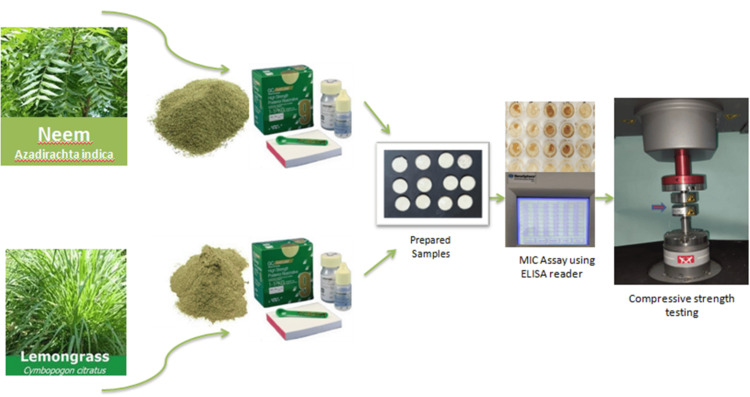
Graphic overview. MIC = minimal inhibitory concentration; ELISA = enzyme-linked immunosorbent assay

This procedure involved mixing the powder and liquid until reaching a paste-like texture, which was then poured into a silicone mold. Following a 30-minute interval, the specimens were extracted from the mold and placed in appropriately labeled containers. Subsequently, they were stored in an incubator at 37°C for 24 hours before being subjected to examinations to evaluate their antimicrobial properties. All samples were prepared by a single trained operator under room temperature conditions of 23°C (Figure 3).

Bacterial strain and inoculum preparation

Strains of *Streptococcus mutans* and *Lactobacillus acidophilus* were procured from the Department of Microbiology, cultured on Mueller-Hinton agar, and subsequently transferred to Mueller-Hinton broth. After 24 hours of incubation at 37°C, the bacterial suspensions were standardized to a concentration of 1.5 × 10^8^ CFU.

Minimal inhibitory concentration assay

To evaluate the antimicrobial efficacy of both modified and unmodified GIC, standard strains of *Streptococcus mutans* and *Lactobacillus *were employed. Each group comprised 12 specimens. At first, we prepared Mueller-Hinton agar broth and then added 200 µL to all the wells within the groups. Bacterial suspensions of both *Streptococcus mutans* and *Lactobacillus acidophilus *were introduced into the wells, each containing approximately 5 × 10^5^ CFU/mL. Minimal inhibitory concentration (MIC) assays were individually performed for the two modified groups (neem and lemongrass). The samples were placed in suitable conditions for varying durations (one to four hours) during the incubation period. An enzyme-linked immunosorbent assay reader was utilized to calculate the percentage of cell death at specific time points by measuring absorbance at a wavelength of 540 nm.

Compressive strength evaluation

**Figure 4:**
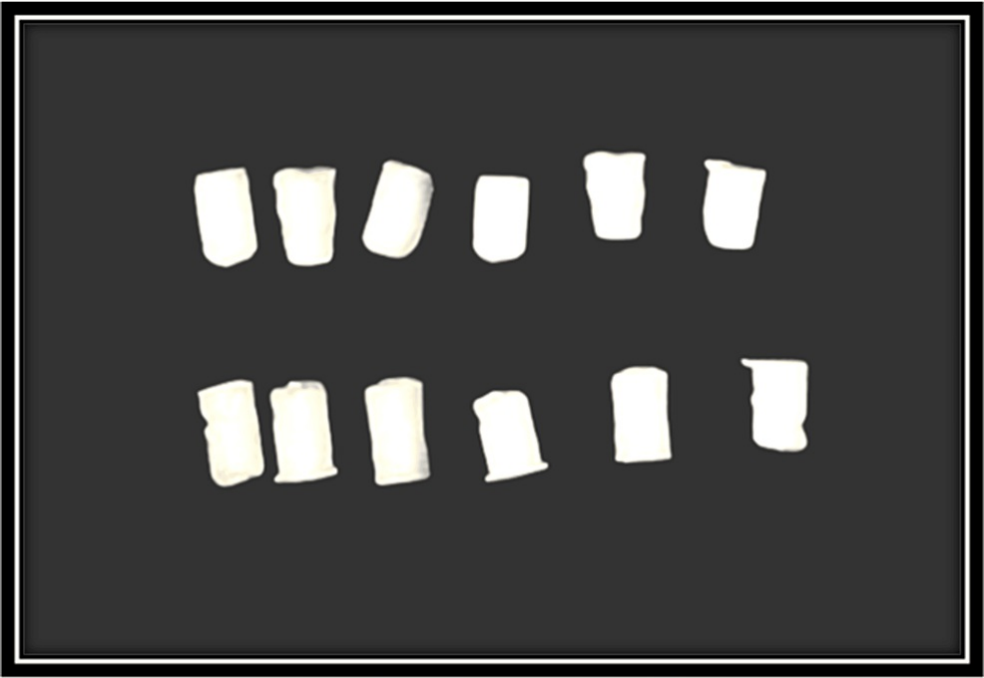
Specimens for testing compressive strength.

The compressive strength evaluation adhered to ISO 9917-1:2007 standards. Within each group, 12 specimens were prepared using cylindrical molds with dimensions of 4.0 mm in diameter and 6.0 mm in height (Figure 4).

After placing the materials in the molds and ensuring a smooth surface, the specimens were allowed to undergo the setting process for one hour. Subsequently, they were immersed in deionized water and stored for 24 hours. Any specimens found with imperfections were excluded from the study. The specimens were positioned vertically within the Universal Testing Machine (Instron ElectroPlus E3000). A compressive force was applied along the extended axis of the specimens at a rate of 0.5 mm/minute until they reached the point of fracture. The maximum force at which the specimens broke was noted, and this value was used to calculate the compressive strength in megapascals (MPa). The testing protocol involved applying compressive force using a 5 kN load cell.

Statistical analysis

The collected data were entered into a Microsoft Excel (Microsoft Corp., Redmond, WA, USA) spreadsheet and subjected to statistical analysis using SPSS version 24 (IBM Corp., Armonk, NY, USA). Descriptive analysis and repeated-measures analysis of variance (ANOVA) were employed to determine the mean MIC values. Comparison of compressive strength among the groups was conducted through a one-way ANOVA, followed by Tukey’s post hoc test for pairwise comparisons. The significance level was set at p-values ≤0.05, with 95% confidence intervals considered.

## Results

Antimicrobial activity

**Figure 5:**
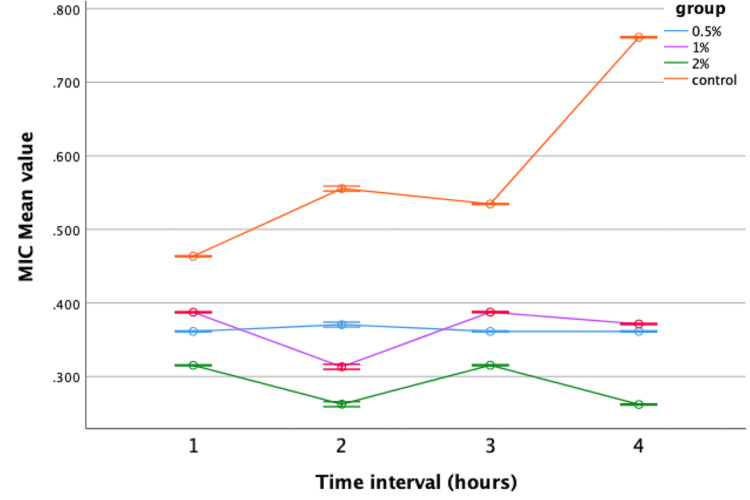
Antimicrobial efficacy of neem-modified GIC against Streptococcus mutans. MIC = minimal inhibitory concentration; GIC = glass ionomer cement

**Figure 6:**
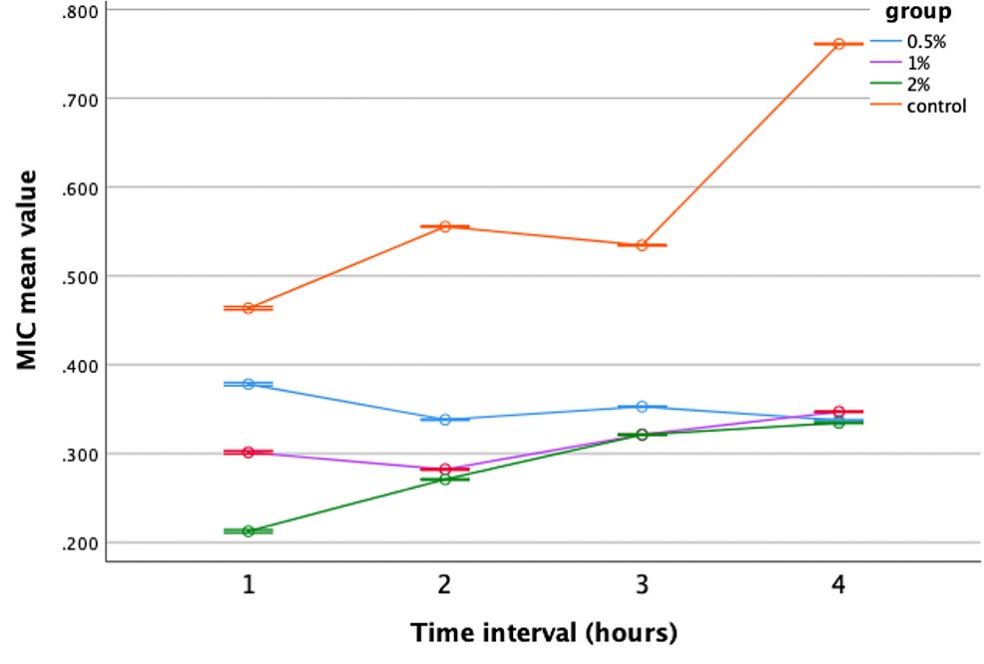
Antimicrobial efficacy of lemongrass-modified GIC against Streptococcus mutans. MIC = minimal inhibitory concentration; GIC = glass ionomer cement

In the MIC assay, neem-modified GIC demonstrated significant antimicrobial efficacy against *Streptococcus mutans* and *Lactobacillus* at all concentrations (0.5%, 1%, 2%) compared to the control group. The 2% concentration exhibited the most promising results, with the lowest mean value recorded in the fourth hour. Similarly, lemongrass-modified GIC showed improved efficacy against *Streptococcus mutans* compared to the control at concentrations of 0.5%, 1%, and 2%. However, no significant difference in antimicrobial activity was observed against *Lactobacillus* compared to the control. Repeated-measures ANOVA was used to assess the antibacterial efficacy of neem and lemongrass-modified GIC groups against *Streptococcus mutans* and *Lactobacillus*. Both groups demonstrated superior performance against *Streptococcus mutans,* with statistically significant results compared to the control group (Figures 5, 6).

**Figure 7:**
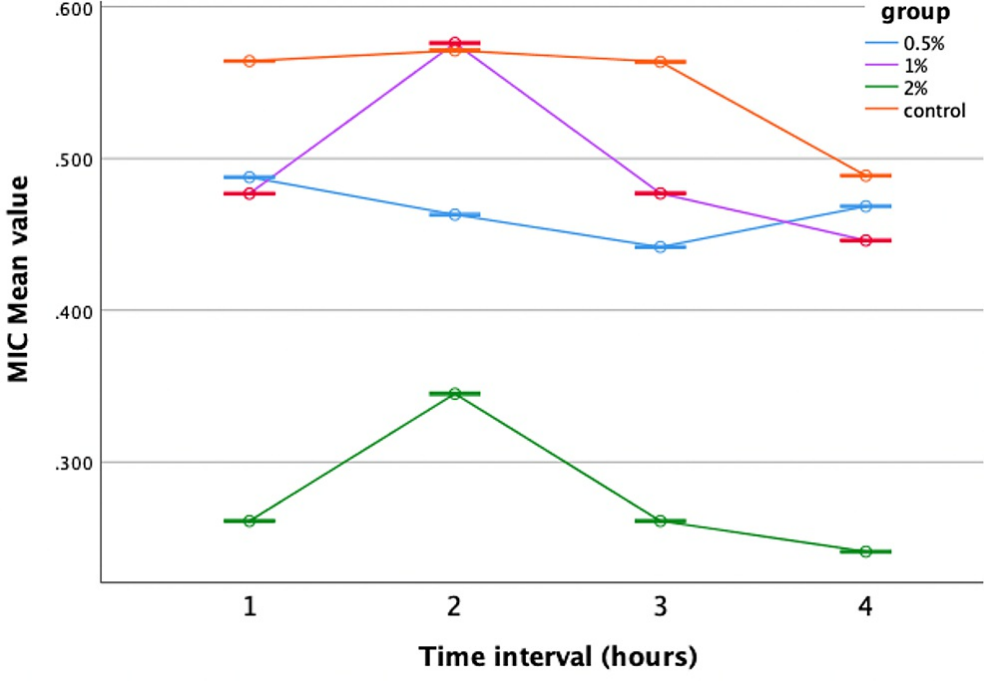
Antimicrobial efficacy of neem-modified GIC against Lactobacillus. MIC = minimal inhibitory concentration; GIC = glass ionomer cement

**Figure 8:**
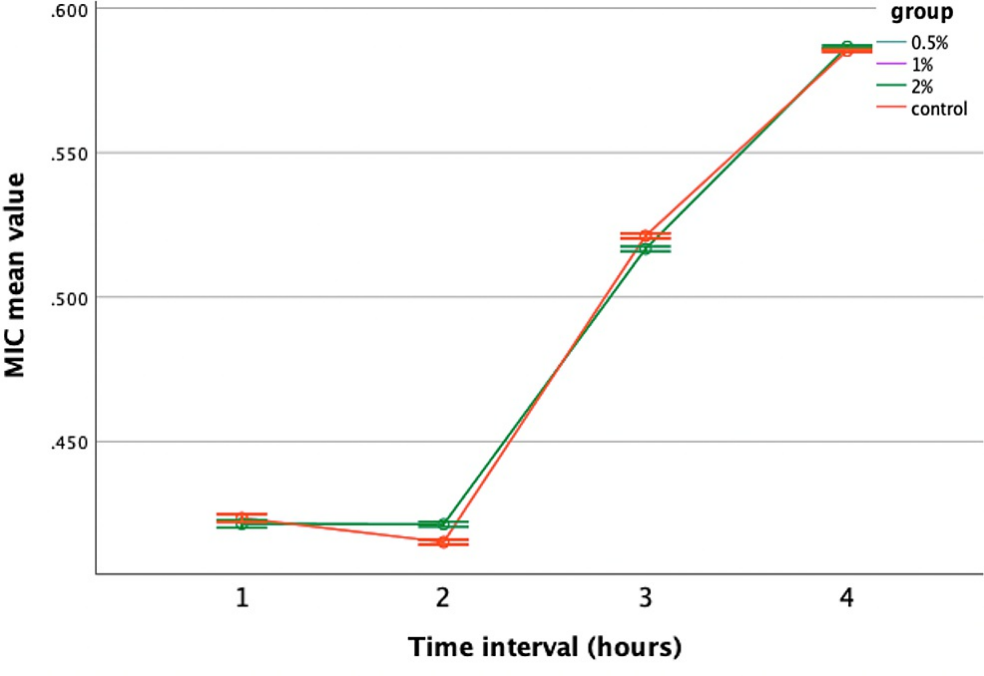
Antimicrobial efficacy of lemongrass-modified GIC against Lactobacillus. MIC = minimal inhibitory concentration; GIC = glass ionomer cement

However, when evaluated against *Lactobacillus*, only neem-modified groups showed greater performance compared to the control, while lemongrass-modified groups displayed comparable results with no statistically significant variances (Figures 7, 8).

In the Tukey honestly significant difference multiple comparison test, there was no significant distinction between the 0.5% and 1% neem-modified groups when tested against *Streptococcus mutans*. However, the 2% concentration exhibited significantly greater antibacterial activity than any other concentration. Additionally, there was a significant difference in antibacterial activity between the neem-modified groups and the control group (p < 0.05) for both *Streptococcus mutans* and *Lactobacillus *(Table 1).

**Table 1:** Pairwise comparison of antimicrobial efficacy of neem-modified GIC on Streptococcus mutans and Lactobacillus. P-value was significant at 0.05. The p-value was derived from multiple comparisons Tukey HSD test. GIC = glass ionomer cement; HSD = honestly significant difference

Pairwise comparison of neem-modified GIC		Mean difference	95% CI		P-value
			Lower	Upper	
Streptococcus mutans	0.5% vs. 1%	0.004	0.002	0.010	0.297
	0.5% vs. 2%	0.074	0.068	0.081	0.001*
	1% vs. 2%	0.079	0.072	0.085	0.001*
	0.5% vs. control	0.094	0.087	0.100	0.001*
	1% vs. control	0.089	0.083	0.096	0.001*
	2% vs. control	0.169	0.162	0.175	0.001*
Lactobacillus	0.5% vs. 1%	0.028	0.028	0.029	0.001*
	0.5% vs. 2%	0.188	0.187	0.188	0.001*
	1% vs. 2%	0.216	0.216	0.217	0.001*
	0.5% vs. control	0.081	0.081	0.082	0.001*
	1% vs. control	0.052	0.052	0.053	0.001*
	2% vs. control	0.269	0.269	0.270	0.001*

For lemongrass-modified GIC, the pairwise analysis revealed a statistically significant difference against *Streptococcus mutans* when compared with the control group (p < 0.05). Furthermore, the 2% concentration showed the maximum antibacterial activity among all concentrations. However, against *Lactobacillus*, no significant difference was observed (p > 0.05). This indicates that there was nearly equivalent antibacterial efficacy between the lemongrass-modified and conventional groups against *Lactobacillus *(Table 2).

**Table 2:** Pairwise comparison of antimicrobial efficacy of lemongrass-modified GIC on Streptococcus mutans and Lactobacillus. P-value was significant at 0.05. The p-value was derived from multiple comparisons Tukey HSD test. GIC = glass ionomer cement; HSD = honestly significant difference

Pairwise comparison of lemongrass-modified GIC		Mean difference	95% CI		P-value
			Lower	Upper	
Streptococcus mutans	0.5% vs. 1%	0.038	0.0375	0.0398	0.001*
	0.5% vs. 2%	0.066	0.065	0.068	0.001*
	1% vs. 2%	0.028	0.026	0.029	0.001*
	0.5% vs. control	0.227	0.225	0.228	0.001*
	1% vs. control	0.265	0.264	0.266	0.001*
	2% vs. control	0.293	0.292	0.295	0.001*
Lactobacillus	0.5% vs. 1%	0.001	-0.004	0.005	1.0
	0.5% vs. 2%	0.001	-0.006	0.003	0.92
	1% vs. 2%	0.001	-0.006	0.003	0.913
	0.5% vs. control	0.004	-0.001	0.009	0.092
	1% vs. control	0.004	-0.001	0.009	0.088
	2% vs. control	0.003	-0.001	0.008	0.275

**Figure 9:**
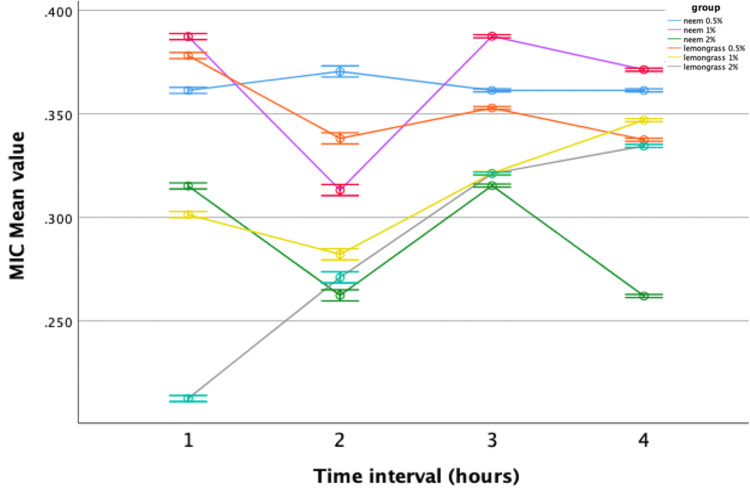
Comparison between neem and lemongrass-modified groups against Streptococcus mutans. MIC = minimal inhibitory concentration

**Figure 10:**
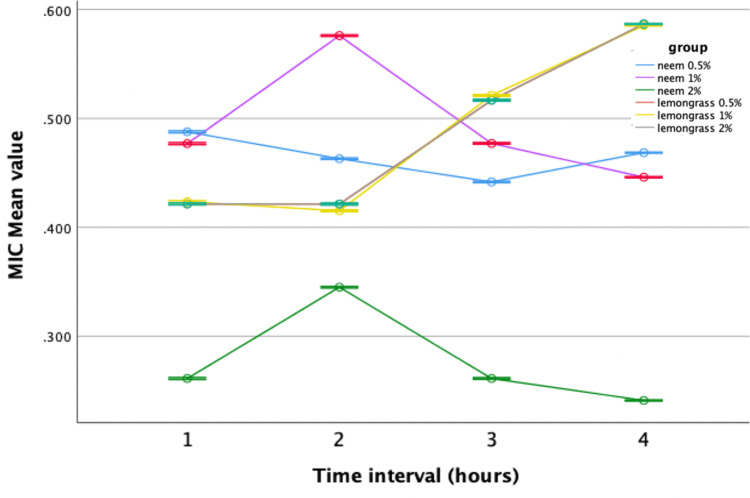
Comparison between neem and lemongrass-modified groups against Lactobacillus. MIC = minimal inhibitory concentration

When comparing neem-modified with lemongrass-modified GIC, 2% neem-modified GIC exhibited superior antimicrobial activity fourth hourly against both *Streptococcus mutans* and *Lactobacillus* (Figures 9, 10).

Compressive strength

The specimens underwent compressive load testing, and the data from the resulting linear graphs was recorded. ANOVA was employed to examine differences in compressive strength among the groups. The examination revealed no statistically significant differences between the modified groups and the control group, as indicated by an F-value of 0.327 and a p-value of 0.921 (p > 0.05) (Table 3).

**Table 3:** Comparison between groups for evaluation of compressive strength. P-value was derived by one-way ANOVA. GIC = glass ionomer cement; ANOVA = analysis of variance

Modified GIC	n	Mean ± SD	SE	95%		df	F-value	P-value
				Lower	Upper			
Neem modified (0.5%)	12	167.80 ± 1.12	0.32	167.08	168.51	6	0.327	0.921
Neem modified (1%)	12	167.76 ± 1.24	0.35	166.97	168.55			
Neem modified (2%)	12	167.97 ± 1.30	0.37	167.14	168.80			
Lemongrass modified (0.5%)	12	167.85 ± 1.02	0.29	167.20	168.50			
Lemongrass modified (1%)	12	168.26 ± 0.91	0.26	167.68	168.85			
Lemongrass modified (2%)	12	167.99 ± 0.75	0.21	167.50	168.47			
Control	12	168.05 ± 0.79	0.22	167.55	168.18			

## Discussion

Early childhood caries, a prevalent dental ailment in children of a young age, remains a global concern [15]. The acid-producing bacterium, *Streptococcus mutans*, in conjunction with different lactobacilli, is known to be associated with caries development [16]. Among these pathogens, *Streptococcus mutans*, which thrives and multiplies in low-pH environments, is considered the most cariogenic. Despite multiple assessments, it has been observed that viable bacteria continue to exist in the remaining affected dentin even after the removal of infected dentin and proper sealing. Modern dentistry commonly employs a class of biomaterials known as glass ionomers. Glass ionomers release fluoride, which aids in the remineralization of softened dentin and reduces the presence of lingering bacteria in cavities [17]. However, their ability to inhibit a wide range of bacteria is limited. Therefore, existing literature supports the therapeutic benefits of combining glass ionomers with antimicrobial agents such as chlorhexidine and antibiotics. Nevertheless, this combination often leads to a compromise in the material’s strength properties [18]. *Azadirachta indica*, commonly known as neem, and *Cymbopogon citratus*, commonly known as lemongrass, are acknowledged for their multifaceted qualities. Therefore, to enhance the properties of GIC, neem, and lemongrass-modified GIC were assessed in this study. Instead of using chemical agents to create a modified GIC, an herbal extract with a history of safety and effectiveness was used in this investigation. Although incorporating an antibacterial agent into restorative materials may pose a potential drawback concerning their physical characteristics, in this study, it was proved that by combining neem extract with GIC and lemongrass with GIC, a new dental material with direct inhibitory characteristics against the chosen bacteria without compromising its strength was generated, according to the results.

Divya et al. assessed the physical characteristics of neem-modified ART restorative materials. Their findings, which demonstrated potential antibacterial effectiveness, align with our study’s results. They also reported greater compressive strength, while in our study, the compressive strength was equivalent to that of conventional GIC, possibly due to variations in the materials used [19]. In our study, neem-modified GIC showed better antimicrobial efficacy against *Streptococcus mutans* and *Lactobacillus *at all concentrations against the control. This might be attributed to the primary active component of neem, azadirachtin, known for its potent antimicrobial properties. Furthermore, other phytochemical components such as nimbidin, nimbin, nimbolide, and azadirachtin play a role in the antibacterial activity [20]. Upon comparison between lemongrass-modified GIC and unmodified GIC, the former demonstrated superior antimicrobial properties. It showed a significant difference in its effectiveness against *Streptococcus mutans*, but no significant difference when tested against *Lactobacillus*. In a prior study by Manvitha et al., it was observed that *Cymbopogon citratus* oil exhibited antibacterial, antifungal, and other medicinal properties [21]. Tofino et al. also tested the antibacterial effects of *Cymbopogon citratus* oil against *Streptococcus mutans* [12], which is in accordance with our study. Syed et al. reported that lemongrass leaf extracts were highly effective in controlling various pathogenic microorganisms [22]. Additionally, Oliveira et al. proved the antimicrobial activity of *Cymbopogon citratus *oil against *Lactobacillus acidophilus*, *Streptococcus mitis*, and *Streptococcus mutans* [23]. Akula et al. demonstrated that lemongrass-based mouthwash effectively reduced plaque and gingivitis in children and could be used as an herbal mouthwash [24]. Kemthong et al. found that *Cymbopogon citratus* essential oil exhibited excellent antibacterial activity [25]. In our study, lemongrass extract was incorporated into the restorative material, and its antibacterial activity against *Streptococcus mutans* was assessed, showing the highest level of effectiveness. However, Kusuma et al. concluded that the growth of *Streptococcus mutans* was not influenced by lemongrass stem infusion at different concentrations [26]. This discrepancy might be attributed to the use of stems in their study, whereas, in our study, we employed lemongrass leaves, which contain pronounced phytochemical constituents and bioactive compounds such as alkaloids, flavonoids, tannins, and phenolic compounds. Flavonoids and terpenoids present in lemongrass extract could be the reason behind inhibiting bacterial growth, as terpenoids possess strong antibacterial effects [27]. Elkorashy reported that lemongrass essential oil modification of GIC effectively improved its antibacterial properties, which is consistent with our findings [28].

Dental restorations need to possess adequate mechanical characteristics as they experience different types of stress within the oral cavity during functions, including compressive, tensile, and shear stresses. According to ISO standards, evaluating the physical characteristics of dental restorations involves conducting compressive strength tests, as most of the forces experienced during chewing are compressive. The compressive strength values may vary based on how the materials are mixed, and placed, and the conditions under which measurements are taken. In this study, the pairwise analysis for compressive strength indicated that there was no significant difference between the experimental group and the control group. This finding aligns with a previous study [29], which indicated that the inclusion of antibacterial materials at specified concentrations did not impact the compressive strength properties of GIC, consistent with our results. Another study by Elkorashy mentioned lower compressive strength values when lemongrass essential oil was combined with GIC [28]. This effect could be attributed to the essential oil’s presence within GIC, which may hinder COOH group interactions within the glass ionomer matrix, potentially slowing down the setting reaction and affecting compressive strength. However, the differences observed in that study were not statistically significant, aligning with our findings.

Within the parameters of this in vitro study, it is evident that neem and lemongrass modifications to GIC can effectively enhance antimicrobial properties while maintaining compressive strength; hence, the null hypothesis was rejected. A comparison between neem and lemongrass modifications indicates that neem displayed superior antimicrobial activity against both *Streptococcus mutans* and *Lactobacillus*. Importantly, both the modifications did not show any significant alterations in compressive strength. This suggests that neem-modified GIC holds promise for clinical applications as it can effectively inhibit the growth of caries-causing bacteria such as *Streptococcus mutans* and *Lactobacillus*. This approach may have practical applications in the treatment of individuals with dental caries. A limitation of the present study is an absence of evaluation for intraoral factors such as masticatory forces, moisture variations, and potential operator discrepancies; therefore, further research is warranted to evaluate the long-term stability and practical applicability of this material in a clinical setting.

## Conclusions

Within the constraints of this study, it can be inferred that the neem-modified GIC exhibits commendable antimicrobial properties against *Streptococcus mutans* and *Lactobacillus *while maintaining an equivalent compressive strength compared to conventional GIC. This modification holds promise as a valuable restorative material for use in operative procedures and addressing secondary caries. Further research is needed to assess the material’s long-term durability and its effectiveness in clinical scenarios.
